# Baseline robot-measured kinematic metrics predict discharge rehabilitation outcomes in individuals with subacute stroke

**DOI:** 10.3389/fbioe.2022.1012544

**Published:** 2022-12-06

**Authors:** Michela Goffredo, Stefania Proietti, Sanaz Pournajaf, Daniele Galafate, Matteo Cioeta, Domenica Le Pera, Federico Posteraro, Marco Franceschini

**Affiliations:** ^1^ Department of Neurological and Rehabilitation Sciences, IRCCS San Raffaele Roma, Rome, Italy; ^2^ Unit of Clinical and Molecular Epidemiology, IRCCS San Raffaele Roma, Rome, Italy; ^3^ Department of Human Sciences and Promotion of the Quality of Life, San Raffaele University, Rome, Italy; ^4^ Rehabilitation Department, Versilia Hospital, Camaiore, Italy

**Keywords:** robot-assisted therapy, stroke, motor recovery, upper extremity, kinematics, biomarkers, predictors

## Abstract

**Background:** The literature on upper limb robot-assisted therapy showed that robot-measured metrics can simultaneously predict registered clinical outcomes. However, only a limited number of studies correlated pre-treatment kinematics with discharge motor recovery. Given the importance of predicting rehabilitation outcomes for optimizing physical therapy, a predictive model for motor recovery that incorporates multidirectional indicators of a patient’s upper limb abilities is needed.

**Objective:** The aim of this study was to develop a predictive model for rehabilitation outcome at discharge (i.e., muscle strength assessed by the Motricity Index of the affected upper limb) based on multidirectional 2D robot-measured kinematics.

**Methods:** Re-analysis of data from 66 subjects with subacute stroke who underwent upper limb robot-assisted therapy with an end-effector robot was performed. Two least squares error multiple linear regression models for outcome prediction were developed and differ in terms of validation procedure: the Split Sample Validation (SSV) model and the Leave-One-Out Cross-Validation (LOOCV) model. In both models, the outputs were the discharge Motricity Index of the affected upper limb and its sub-items assessing elbow flexion and shoulder abduction, while the inputs were the admission robot-measured metrics.

**Results:** The extracted robot-measured features explained the 54% and 71% of the variance in clinical scores at discharge in the SSV and LOOCV validation procedures respectively. Normalized errors ranged from 22% to 35% in the SSV models and from 20% to 24% in the LOOCV models. In all models, the movement path error of the trajectories characterized by elbow flexion and shoulder extension was the significant predictor, and all correlations were significant.

**Conclusion:** This study highlights that motor patterns assessed with multidirectional 2D robot-measured metrics are able to predict clinical evalutation of upper limb muscle strength and may be useful for clinicians to assess, manage, and program a more specific and appropriate rehabilitation in subacute stroke patients.

## Introduction

Most stroke survivors experience upper limb motor impairments that negatively influence Activities of Daily Living (ADL) (Nichols-Larsen et al., 2005). Over the past two decades, robotic devices have been shown to provide intensive and highly repeatable therapy, enrich the sensorimotor experience, and offer customizable and repeatable support during treatment (Mehrholz et al., 2018; Morone et al., 2020; Gandolfi et al., 2021). In particular, Mehrholz et al. evidenced the efficacy of upper limb Robot-assisted Therapy (ulRT) in improving ADL, arm function, and arm muscle strength (Mehrholz et al., 2018) in stroke patients.

Recently, robots have been recognised not only as a rehabilitation device but also as a measurement tool, suggesting that they can provide a standardized and objective measure of a patient’s motor control and improve research knowledge on treatment effects and stroke recovery (Agrafiotis et al., 2021). In this regard, studies on ulRT have analyzed the Robot-Measured Kinematic (RMK) data to assess ulRT-induced biomechanical changes and patient progress over time (Dipietro et al., 2011; Balasubramanian et al., 2012; Panarese et al., 2012; Mazzoleni et al., 2013; Tran et al., 2018; Goffredo et al., 2019). Furthermore, RMK data have also been shown to be able to capture relevant aspects of goal-directed movements that may reveal pathological motor synergies in stroke survivors (Dipietro et al., 2011; Panarese et al., 2012; Goffredo et al., 2019). RMK metrics were also found to correlate with motor impairment as measured by Fugl-Meyer upper limb assessment (Colombo et al., 2005; Duret et al., 2016) and to be representative of pathological motor synergies when different directions of movement were analyzed (Panarese et al., 2012; Goffredo et al., 2019). The latter is in accordance with the kinematic approach to identify how the central nervous system represents and implements the motor control strategies necessary to obtain the movements in stroke patients (Micera et al., 2005). RMK data were also processed to predict the clinical assessment outcomes (Krebs et al., 2014; Duret et al., 2019; Agrafiotis et al., 2021; Goffredo et al., 2021; Grimm et al., 2021; Moretti et al., 2021), considering the importance of biomarkers of neurorehabilitation outcomes for evidence-based practice (Langhorne et al., 2009; Scott and &Dukelow, 2011; Duret et al., 2015; Franceschini et al., 2018). In this context, the Predict REcovery Potential (PREP2) tool is the predominant predictor of upper limb functional outcomes from clinical assessment, Magnetic Resonance Imaging (MRI) and Transcranial Magnetic Stimulation (TMS) biomarkers, with an impact on rehabilitation planning and realistic treatment goal setting (Stinear et al., 2017). Although PREP2 predicts correctly approximately 70% of patients, the major limitation is that TMS is not readily available in many clinical settings (Connell et al., 2021). Therefore, in rehabilitation hospitals equipped with robots for ulRT, an alternative, ecological robot-based method to predict rehabilitation outcome could be the analysis of RMK data at baseline. However, to the best of our knowledge there is no evidence in literature on the RMK-based predictors of clinical outcomes at discharge that account for motor synergies at baseline.

Considering the importance of quantitative indicators of upper limb function, Krebs *et al.* and Moretti *et al.* found that RMK metrics from a 2D robot can be biomarkers of clinical outcomes registered on the same day (Krebs et al., 2014; Moretti et al., 2021). Their findings were consistent with those of Grimm et al. who analyzed exoskeleton-based kinematics (Grimm et al., 2021). On the other hand, Agrafiotis *et al.*, developed RMK-based models of clinical outcomes with the aim of removing inter- and intra-rater variability and reducing the sample size in stroke clinical trials (Agrafiotis et al., 2021). To our knowledge, only Duret *et al.* predicted upper limb recovery at the end of ulRT (Duret et al., 2019). They found that selected RMK parameters, calculated from the total end-effector trajectory did not predict the upper limb Fugl-Meyer Assessment. In our previous paper (Goffredo et al., 2021), we analyzed RMK metrics calculated from reaching movements with different directions. Specifically, the movement path error, the mean movement speed, and the number of speed peaks of each point-to-point trajectory were analyzed. Then, a generalized linear analysis was applied to estimate the relationships between the RMK measures at baseline and the (clinical and RMK) data at the end of ulRT, considering each direction of movement separately. The analysis revealed that a subset of the RMK metrics was correlated with the probability of rising one class in the Motricity Index at the end of ulRT. Our multidirectional 2D analysis of RMK metrics showed that pre-treatment kinematic data are representative of pathological motor synergies in stroke survivors, i.e., the ability to perform movements of shoulder (ab-adduction, internal and external rotation) and elbow (flexion-extension) is representative of flexor synergy in stroke patients (Goffredo et al., 2021). However, despite the importance of predicting rehabilitation outcomes for clinician decision-making and treatment optimization, there is a paucity of literature on post-ulRT rehabilitation outcomes based on patients’ upper limb abilities and motor synergies at baseline.

The aim of this study is to re-analyze the retrospective data from our previous study to develop a multidirectional 2D RMK-based predictive model for rehabilitation outcome (i.e., muscle strength assessed by the Motricity Index of the affected upper limb) at the end of ulRT. Potential predictors included patient demographics, stroke characteristics, and pre-treatment RMK metrics calculated considering upper limb movements which are representative of the stroke flexor synergy. In order to reliably predict the discharge rehabilitation outcome, we compared two least squares error multiple linear regression models that differ in terms of the output validation procedure.

## Materials and methods

This is a re-analysis of data acquired and processed by the IRCCS San Raffaele Roma (Rome, Italy) in an observational retrospective study (Ethical approval no. 06/17; 22/02/2017) on stroke inpatients who underwent ulRT in addition to the conventional therapy (Goffredo et al., 2021).

### Patients and treatments

Sixty-six stroke patients who were trained for 20 sessions (5 times/week; 45 min per session) with the In Motion 2 robot (Bionik Laboratories, Watertown, MA, United States), were included in the study. The persons were inpatients admitted to the IRCCS San Raffaele Roma (Rome, Italy) between January 2011 and December 2017 who satisfied the following inclusion criteria: age between 18 and 80 years; first event of unilateral hemiparetic stroke; subacute phase (RT started within 30 ± 7 days post stroke); upper limb Chedoke-McMaster scores between 2 and 5; Motricity Index affected upper limb<100; ulRT for 20 sessions. Subjects were excluded from the study if they had bilateral impairment; chronic phase; ulRT for less than 20 sessions; interruption of the ulRT for more than three consecutive days; presence of other severe medical conditions; incomplete data in the database. More detailed information on the ulRT conducted by the patients is available in the previous papers of the authors (Goffredo et al., 2019; Goffredo et al., 2021).

### Data collection and feature extraction

The following demographic and clinical data have been collected at baseline: age, gender, affected side, stroke onset time, and etiology. Moreover, the following clinical outcomes were recorded before (T1) and after (T2) the ulRT: Motricity Index of the affected Upper Limb (MI_UL_) (Bohannon, 1999), Motricity Index sub-item assessing the elbow flexion (MI_ELBOW_), and Motricity Index sub-item assessing the shoulder abduction (MI_SHOULDER_). The MI_UL_ is a discrete scale measuring the muscle strength of the paretic upper extremity. Three actions were separately assessed: pinch grasp, elbow flexion, and shoulder abduction. Each action was scored (0–33) with a MI sub-item composed of six classes, as defined by Wade (Wade, 1989). The total upper extremity score (MI_UL_) was calculated by adding one to the sum of the three sub-items (maximum possible score = 100). The MI sub-item related to the pinch grip was not considered in this study because the InMotion2-based ulRT typically involves the elbow and shoulder joints.

**FIGURE 1:**
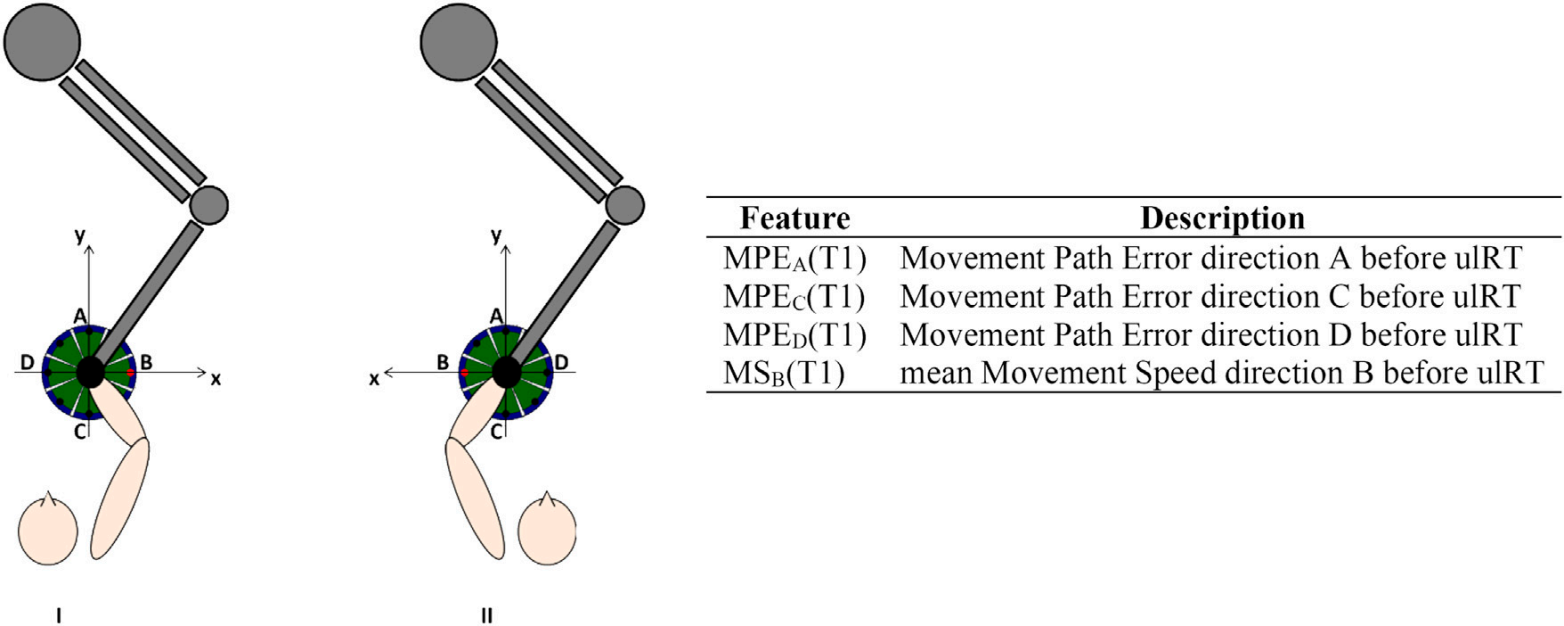
Experimental setup, reference system in case of right (I) or left (II) affected limb, and description of the features considered for the models’ development.

The RMK metrics were calculated from the trajectories (200 Hz) recorded by the robot, considering each movement direction separately (Goffredo et al., 2021). Figure 1 shows the reference system (coinciding with the lesion side) used to calculate the RMK metrics. Considering the results of our previous analysis of kinematic biomarkers for upper-limb motor recovery (Goffredo et al., 2021), we extracted the features that were correlated with muscle strength at discharge, i.e.: Movement Path Error direction A (MPE_A_), direction C (MPE_C_), and direction D (MPE_D_); and mean Movement Speed direction B (MS_B_).

The MPE is a measure of accuracy (the value is 0 if the trajectory lies exactly on a straight line connecting the initial and the final target): it is computed as the mean value of the distance between each point of the actual path travelled by the subject from the ideal one (i.e., the straight line connecting the central target and the peripheral one). Since the considered peripheral targets are along the x and y axes, the MPE computation is the following:

$$MPE=\left\{ \begin{array}{cc} \frac{1}{N}\overset{N}{\underset{k=1}{\sum }}\left|y\left[k\right]\right| & direction\;D\\ \frac{1}{N}\overset{N}{\underset{k=1}{\sum }}\left|x\left[k\right]\right| & directions\;A\;and\;C \end{array}\right.$$ (1)

where N is the number of samples for each trajectory, identified by the coordinates x[k] and y[k] in the xy plane. The MS has been computed from the discrete-time velocity signals v_x_[k] and v_y_[k] along the x and y axes, respectively as the mean value of the resultant velocities in the xy plane:

$$MS=\frac{1}{N}\overset{N}{\underset{k=1}{\sum }}\sqrt{{\left(v_{x}\left[k\right]\right)}^{2}+{\left(v_{y}\left[k\right]\right)}^{2}}$$ (2)

All RMK metrics were recorded at T1 and showed good test-retest reliability (Koeppel and Pila, 2020). Prior to modeling, age, stroke onset time, and RMK metrics have been standardized by subtracting the mean and scaling to unit variance. Figure 1 includes the list of features used for model development.

### Statistical analysis and predictive models

All statistical analyses were performed using the R statistical package system v. 4.2.0 (R Foundation for Statistical Computing, Austria). A significance level of 5% (*p*-value ≤ 0.05) was assumed. Demographic and clinical data were reported with frequencies and percentages if they were categorical variables, while continuous variables were expressed with mean and standard deviation, median, and interquartile range.

The study compared two types of outcome prediction models that differed in terms of the validation procedure. The first model was the least squares error multiple linear regression model with the Split Sample Validation procedure (SSV model). The second model was the least squares error multiple linear regression with the Leave-One-Out Cross-Validation procedure (LOOCV model). In both models, the output**s** were MI_ELBOW_ (T2), MI_SHOULDER_ (T2), and MI_UL_ (T2) discharge scores (adjusted for age, sex, stroke onset time, and clinical assessment scores admission), whereas the inputs were the RMK metrics at admission.

For the SSV models, 75% of the data were randomly separated for model training, whereas the remaining 25% were set aside for model validation (Bosecker et al., 2010). The Chi-square test for categorical variables, and ANalysis Of VAriance (ANOVA) for continuous variables when normally distributed, and otherwise Mann Whitney test, confirmed that the training and validation data sets were not significantly different. Moreover, the data set was randomly divided into four groups (25% data each), and the Kruskal Wallis test (Bonferroni’s correction) confirmed that there was no significant difference (*p*-value>0.05) between any combination of these groups in training and validation sets.

For the LOOCV models, one of the 66 records was removed, and the remaining were used to build the model. The resulting model was then used to make predictions about the record set aside (Bishop, 1995). This was repeated for each of the 66 cases.

Each prediction model was described by the percentage of variance explained (R^2^), the adjusted R^2^ (R_adj_^2^), the Mean Absolute Error (MAE), Root Means Squared Error (RMSE), and normalized RMSE (RMSE_n_). The normalized MAE (MAE_n_) was calculated to compare the performance of the model for the different dependent variables.

Results were assessed by correlation between therapist-assigned values and corrected predicted values (Spearman’s rank correlation coefficient, r, and associated *p* values). Since the MI_UL_ is a discrete variable, each predicted value was corrected using a nearest neighbor procedure by assigning the score with the minimum Euclidean distance from the valid scores. Results were assessed by correlation between therapist-assigned scores and corrected predicted scores (Spearman’s rank correlation coefficient, r, and associated *p*-values). The strength of the correlations calculated in the analyses was interpreted as follows; |r|=0–0.3 very weak, |r|=0.31–0.5 weak, |r|=0.51–0.7 moderate, and |r|=0.71–1.0 strong (Moore et al., 2013).

### Ethical considerations

Since March 2012, the Italian Data Protection Authority (Garante per la protezione dei dati personali) declared that IRCCS (Istituto di Ricovero e Cura a Carattere Scientifico - Institute for scientific research and healthcare) is authorized to perform retrospective studies without the approval of the local Ethical Committee, and mandatory formal communication is sufficient. Such communication relative to this study was registered by the Ethical Committee of the IRCCS San Raffaele Roma (Rome, Italy) on 22/02/2017 (code number: 06/17).

## Results

In this study, we compared outcome predictors obtained by two modeling procedures differing from one another by output validation.

### Split sample validation models

Table 1 shows the patient’s demographics and clinical characteristics at baseline: data are shown for all recruited subjects, training set, and validation set separately. No significant differences (*p*-value>0.05) were registered between the training and the validation sets.

**TABLE 1:** Sample characteristics at baseline (T1).

	All data (*n* = 66)	Training set (*n* = 50)	Validation set (*n* = 16)
Age (years)	64.97 ± 12.75	64.84 ± 13.55	65.38 ± 10.26
Sex, male/female	44 (66.7%)/22 (33.3%)	32 (64.0%)/18 (36.0%)	12 (75.0%)/4 (25.0%)
Side, right/left	39 (59.1%)/27 (40.9%)	27 (54.0%)/23 (46.0%)	12 (75.0%)/4 (25.0%)
Stroke onset time (days)	15.27 ± 18.07	12.88 ± 7.97	22.75 ± 33.59
Etiology, ischemic/hemorrhagic	47 (71.2%)/19 (28.8%)	34 (68.0%)/16 (32.0%)	13 (81.3%)/3 (18.8%)
MI_ELBOW_(T1)	14.0 (9.0–19.0)	14.0 (9.0–19.0)	16.5 (2.25–25.0)
MI_SHOULDER_(T1)	14.0 (9.0–19.0)	14.0 (9.0–19.0)	16.5 (2.25–25.0)
MI_UL_(T1)	42.0 (19.0–62.0)	40.5 (19.0–58.75)	50.5 (8.25–76.0)

Data are shown as N (%), mean ± SD, or median (IQR). Motricity Index affected elbow flexion (MI_ELBOW_); Motricity Index affected shoulder abduction (MI_SHOULDER_); Motricity Index affected Upper Limb (MI_UL_); before ulRT (T1).

Predictive models for muscle strength at T2 of each clinical outcome were developed using the least squares error multiple linear regression on the training set. In all models, the movement path errors in the C (MPE_C_) and D (MPE_D_) directions were significant predictors (*p*-value<0.05). Specifically, MPE_C_ provided larger contribution (MI_ELBOW_: β = −5.16; MI_SHOULDER_: β = −5.02; MI_UL_: β = −13.52) than MPE_D_ (MI_ELBOW_: β = 3.37; MI_SHOULDER_: β = 3.37; MI_UL_: β = 8.26).

The training and validation results of the SSV models for estimating the MI_ELBOW_ (T2), MI_SHOULDER_ (T2), and MI_UL_ (T2) are shown in Table 2. All correlations were significant at *p*-value < 0.05. The residual plots had no significant patterns, indicating that no underlying trends in the data were missed and that the model was fit. The MI_ELBOW_ registered a moderate correlation in the training set (R^2^ = 0.683), but a weak one in the validation set (R^2^ = 0.481). This decrease is more remarkable in MI_SHOULDER_, which is characterized by an R^2^ of 0.640 in the training set and a very weak level of correlation in the validation one (R^2^ = 0.200). The MI_UL_ showed the highest R^2^ value in both datasets. The resulting models explained 64%–76% of the variance in discharge scores, in the training set. For predicting the clinical outcomes of a patient in the validation set, the average error was 6.098 points for the MI_ELBOW_ model (range 0–33), 8.357 points for the MI_SHOULDER_ model (range 0–33), and 17.265 points for the MI_UL_ model (range 1–100).

**TABLE 2:** SSV predictive models for the clinical outcomes (MI_ELBOW_, MI_SHOULDER_, MI_UL_) at the end of ulRT (T2). Results of the training and validation sets are depicted separately.

	SSV models							
	Training set (*n* = 50)				Validation set (*n* = 16)			
	RMSE	RMSE_n_ (%)	R^2^ (R_adj_^2^)	MAE (MAE_n_)	RMSE	RMSE_n_ (%)	R^2^ (R_adj_^2^)	MAE (MAE_n_)
MI_ELBOW_(T2)	5.547	17	0.683 (0.622)	4.234 (18%)	8.186	25	0.481 (0.35)	6.098 (24%)
MI_SHOULDER_(T2)	5.294	16	0.640 (0.570)	4.372 (19%)	11.588	35	0.200 (0.14)	8.357 (35%)
MI_UL_(T2)	13.859	14	0.765 (0.719)	10.400 (15%)	21.650	21.8	0.628 (0.54)	17.265 (24%)

Motricity Index affected elbow flexion (MI_ELBOW_); Motricity Index affected shoulder abduction (MI_SHOULDER_); Motricity Index affected Upper Limb (MI_UL_); Root Mean Square Error (RMSE); normalized Root Mean Square Error (RMSE_n_);percentage of variance explained (R^2^); adjusted percentage of variance explained (R_adj_^2^); Mean Absolute Error (MAE); normalized Mean Absolute Error (MAE_n_); after ulRT (T2).

**FIGURE 2:**
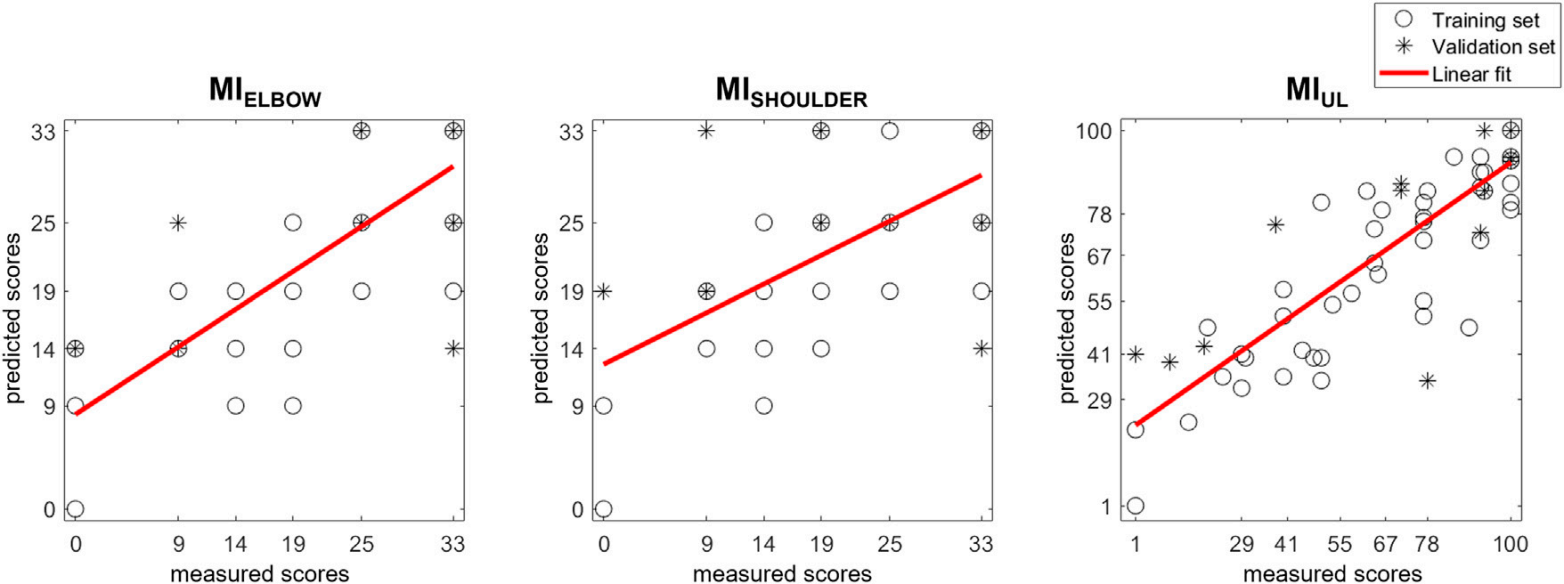
Scatter plot of MI scores prediction with SSV models. Each patient’s actual score was plotted together with the predictions generated by the models. Training and validation sets are depicted separately. The results of a linear polynomial fitting are also shown.

To illustrate the MI score predictions, the actual score of each patient was plotted together with the predictions generated by the models (Figure 2). The correlation between the therapist-assigned scores and predicted scores was moderate for MI_ELBOW_ (r = 0.794; *p*-value < 0.001) and MI_SHOULDER_ (r = 0.627; *p*-value < 0.001), and strong for MI_UL_ (r = 0.839; *p*-value < 0.001).

### Leave-one-out cross-validation models

The following LOOCV predictive models of discharge MI_ELBOW_, MI_SHOULDER_, and MI_UL_ were obtained:

$${\hat{MI}}_{ELBOW}\left(T2\right)=12.99-4.89∙{MPE}_{C}\left(T1\right)$$ (3)

$${\hat{MI}}_{SHOULDER}\left(T2\right)=13.70-4.26∙{MPE}_{C}\left(T1\right)$$ (4)

$${\hat{MI}}_{UL}\left(T2\right)=32.50-11.99∙{MPE}_{C}\left(T1\right)$$ (5)

The generalizability of each model was evaluated by testing its ability to predict scores of patients who were not involved in the development of the model (Table 3). In all models, the MPE_C_ (i.e., the error of the trajectory towards the body) was the significant predictor, and all correlations were significant (*p*-value<0.05). The residual plots did not have any significant correlations. The resulting models explained 46.4%–67.6% of the variance in discharge scores, and the normalized RMSE ranged from 17% to 22%.

**TABLE 3:** Results from the predictive models for the discharge clinical outcomes (MI_ELBOW_, MI_SHOULDER_, MI_UL_)by applying the Leave-One-Out Cross-Validation (LOOCV) procedure.

	LOOCV models			
	RMSE	RMSE_n_ (%)	R^2^ (R_adj_^2^)	MAE (MAE_n_)
MI_ELBOW_(T2)	6.768	20	0.676 (0.631)	5.165 (22%)
MI_SHOULDER_(T2)	7.177	22	0.600 (0.545)	5.680 (24%)
MI_UL_(T2)	17.103	17	0.753 (0.719)	13.487 (20%)

The “new patients” MAE is the averaged error across all left-out subjects during the LOOCV procedure. Abbreviations:Motricity Index affected elbow flexion (MI_ELBOW_); Motricity Index affected shoulder abduction (MI_SHOULDER_); Motricity Index affected Upper Limb (MI_UL_); Root Mean Square Error (RMSE); normalized Root Mean Square Error (RMSE_n_);percentage of variance explained (R^2^); adjusted percentage of variance explained (R_adj_^2^); Mean Absolute Error (MAE); normalized Mean Absolute Error (MAE_n_); after ulRT (T2).

**FIGURE 3:**
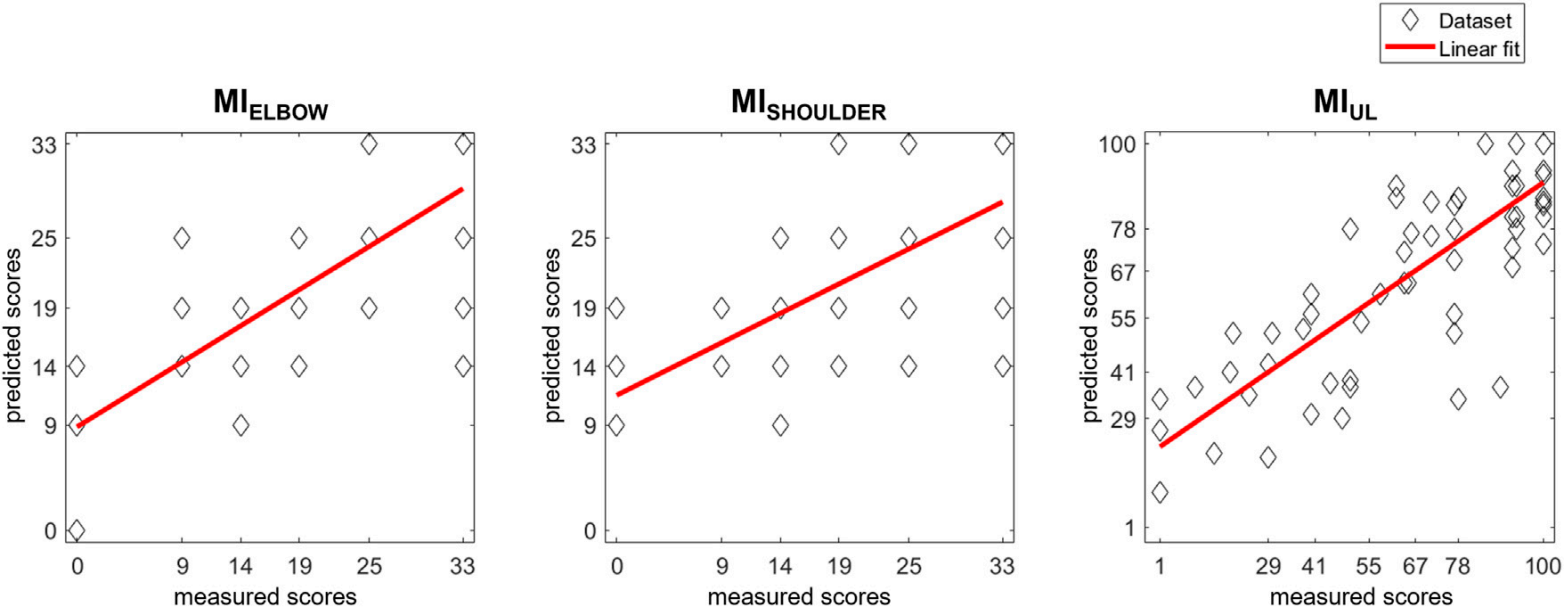
Scatter plot of MI scores prediction with LOOCV models. Each patient’s actual score was plotted together with the predictions generated by the models. The results of a linear polynomial fitting are also shown.

Figure 3 depicts the actually measured score with the predicted ones generated by the models. The correlation coefficients between the therapist-assigned scores and the model’s output evidences a significant correlation in all outcomes: MI_ELBOW_ (r = 0.747; *p*-value < 0.001), MI_SHOULDER_ (r = 0.653; *p*-value < 0.001), and MI_UL_ (r = 0.813; *p*-value < 0.001).

## Discussion

Predicting rehabilitation outcomes based on pre-treatment characteristics would be of great benefit to clinicians in setting realistic rehabilitation goals, personalizing treatment activities, and supporting patient discharge in an appropriate setting (Stinear et al., 2017). In the present article, we examined the predictive abilities of two types of multiple linear regression models to reliably predict the rehabilitation outcome at discharge using RMK-based features. Specifically, both SSV and LOOCV validation procedures were considered. Since the ability to perform point-to-point reaching movements in different directions is considered in the literature to be representative of different synergies involved in the performance of reaching tasks (Panarese et al., 2012; Goffredo et al., 2019), pre-treatment RMK metrics were considered as predictive features for rehabilitation outcomes at discharge in the models developed in this study, with each direction of movement assessed separately.

The procedure consisted of a re-analysis of 66 inpatients with subacute stroke who were included in a 4-week ulRT and whose data had been examined in our previous studies with different aims (Goffredo et al., 2019; Goffredo et al., 2021). In particular, in Goffredo et al. (Goffredo et al., 2021), a relationship was found between RMK measures at baseline and (clinical and RMK) data at the end of ulRT, considering each direction of movement separately (Goffredo et al., 2021), and revealing that a subset of kinematic parameters was correlated with the probability of rising one class in the Motricity Indexes (when considered as categorical variables). In contrast, in the present study, we developed models to predict Motricity Indexes at discharge from selected RMK features and found that the SSV and LOOCV validation procedures explained 54% and 71% of the variance in clinical scores at discharge respectively. Normalized errors ranged from 22% to 35% in the SSV models, and from 20% to 24% in the LOOCV ones.

Our findings are in agreement with the studies of Agrafiotis *et al.* (Agrafiotis et al., 2021) and Moretti *et al.* (Moretti et al., 2021), although they analyzed chronic stroke patients. Both studies found a significant correlation between RMK metrics and clinical outcomes (upper limb Fugl-Meyer Assessment (Agrafiotis et al., 2021; Moretti et al., 2021), Motor Power (Agrafiotis et al., 2021), NIH stroke scale (Agrafiotis et al., 2021), modified Rankin scale (Agrafiotis et al., 2021), Wolf Motor Function Test (Moretti et al., 2021), Barthel Index (Moretti et al., 2021), and Medical Research Council score (Moretti et al., 2021) demonstrating that traditional stroke assessment scales can be accurately reproduced by robotic measurements. The outcomes of these studies pave the way for the use of RMK data to reduce the sample size needed for future clinical trials on chronic stroke patients (Agrafiotis et al., 2021) and to objectively, quantitatively, and rapidly assess impairments in body function (Moretti et al., 2021). However, most published studies on this topic examined the relationships between technology-based metrics and clinical assessment outcomes measured close in time (e.g., concurrently) (Krebs et al., 2014; Grimm et al., 2021; Olesh et al., 2014; Wang et al., 1109). Conversely, our findings showed that baseline data collected from a rehabilitation robotic device are able to predict clinical outcomes at discharge with statistically significant accuracy. Mostafavi *et al.* (Mostafavi et al., 2013) showed results similar to ours although the RMK metrics derived from an exoskeleton robot and the predicted rehabilitation outcome at discharge was an overall measure of disability in ADL (i.e., the Functional Independence Measure).

Data analysis showed that the significant features for predicting the discharge Motricity Indexes were the errors of the trajectories towards the body, which were strongly influenced by the typical postural patterns of the upper extremity after stroke (Raghavan, 2015): the trajectory towards the body is performed by flexing the elbow and extending the shoulder and is representative of upper limb spastic co-contraction (Bensmail et al., 2010) and the typical pathological flexor strategy of stroke survivors (Hefter et al., 2012; McMorland et al., 2015). In our analysis, the error of the trajectory towards the body was a significant predictor in both SSV and LOOCV models, showing that the less accurate and controlled the trajectory towards the pathological patterns is, the smaller muscle strength, assessed with the Motricity Index, is at the end of the ulRT. Our findings agree partly with Gialanella & Santoro (Gialanella and Santoro, 2015), who showed that at the end of rehabilitation, the motor score of the functional independence measure was lower in patients having at admission, the only flexor synergy of the affected limbs. Similarly, the systematic review by Coupar *et al.* (Coupar et al., 2012) found strong evidence that less impairment at baseline is associated with better upper limb recovery. Conversely, the outcomes of our model are not in accordance with Welmer *et al.* (Welmer et al., 2006) who found that stroke patients with typical pathological synergies had significantly better functioning scores.

In our previous analysis of the data (Goffredo et al., 2021), the error of the trajectory towards the body negatively affects the probability of increasing one class in the discharge Motricity Indexes. However, in the re-analysis of data with least squares multiple linear regression, we found that the error of the trajectory towards the body was a significant predictor of Motricity Indexes, which strongly correlate (r > 0.8 for the MI_UL_) with the therapist-assigned ones (Figures 2, 3). The comparison between the SSV and the LOOCV models showed that the error of the trajectory towards the body was a significant predictor in both models, whereas the same metric towards target D appeared in the SSV models.

In the literature, the most widely recognized predictor of upper limb functional outcome is the PREP2 tool (Stinear et al., 2017; Connell et al., 2021), which is based on clinical, MRI, and TMS biomarkers. Despite its high accuracy, the major characteristic of PREP2 is that TMS is not readily available in many clinical settings. Therefore, in rehabilitation hospitals equipped with robots for ulRT, our ecological, quantitative, objective analysis of baseline RMK data could be a valuable alternative to predict the rehabilitation outcome. Furthermore, since RMK metrics are representative of the ability to perform goal-directed movements, RMK biomarkers are able to predict rehabilitation outcomes according to the motor synergies at the baseline.

This study has the following limitations due to its retrospective design: a limited number of subjects; lack of an ICF-based assessment (considering body function, activity, and participation); and lack of RMK data from able-bodied subjects. Considering the SSV models, the training and validation datasets differed with respect to the time of stroke onset: although no statistical significance was found between groups (*p*-value>0.05), it may have influenced the presence of stereotypic movement synergies. Although the analysis normalized the time of stroke onset before modelling, future studies with a more homogeneous sample of patients would be interesting. The limitation of the study in terms of database size can be partially overcome considering that LOOCV models are more reliable and unbiased than SSV ones (Bishop, 1995) and seem particularly suitable when the dataset is small and an accurate estimation of model performance is required. In addition, the LOOCV models confirmed the outcomes of the SSV procedure with a correlation of up to R^2^ = 0.753 (RMSE_n_ = 17%). However, the future research agenda should consider large longitudinal studies including different categories of robots for upper limb rehabilitation (Gandolfi et al., 2021), evaluating the patients with an ICF-based assessment, and comparing the outcomes with a control group composed of able-bodied subjects.

Nevertheless, the study highlights that in stroke ulRT, the motor patterns assessed with RMK metrics strongly relate to discharge rehabilitation outcome and that the accuracy in performing elbow flexion movement is a significant predictor of outcome. The developed models, thus, are able to predict the clinical assessment of upper limb muscle strength and can be useful to clinicians to assess, manage, and program a more specific and appropriate rehabilitation in subacute stroke patients.

## Conclusion

Multidirectional 2D RMK-based predictive models were developed and validated for discharge rehabilitation outcomes in sixty-six subacute stroke patients who performed ulRT with a planar end-effector robot. Accuracy in performing elbow flexion and shoulder extension movements was found to be a significant predictor of muscle strength at the end of ulRT: patients with a pathological upper limb flexor strategy were less likely to increase muscle strength at discharge.

Since the potential recovery of motor function depends on the synergies that occur after stroke, quantitative and measurable knowledge of upper limb function before initiation of physical therapy could be useful for an accurate and individualized prognosis, allowing more realistic expectations for recovery and helping to set realistic goals with a personalized rehabilitation program. In this respect, the results of this study suggest that subacute stroke patients with a marked flexor strategy tend to have a worse rehabilitation outcome at discharge: in these cases, physical therapy should focus on developing beneficial health synergies and avoid reinforcing pathological patterns.

## Data Availability

The raw data supporting the conclusions of this article will be made available by the authors, without undue reservation.
